# Molecular Research on Muscle Protein and Myopathies

**DOI:** 10.3390/ijms23137098

**Published:** 2022-06-26

**Authors:** Olga Karpicheva

**Affiliations:** Laboratory of Molecular Basis of Cell Motility, Institute of Cytology of the Russian Academy of Sciences, 4 Tikhoretsky Ave., 194064 Saint Petersburg, Russia; olexiya6@yandex.ru

This Special Issue highlights new data on the molecular mechanisms of muscle functioning under normal conditions and cellular dysfunctions. Every muscle cell in skeletal, cardiac, or smooth muscle tissue is characterized by the ability to contract. Muscle contraction is based on the cyclic interaction between actin and myosin and is accompanied by ATP hydrolysis in the active center of the myosin motor domain. The myosin filaments slide relative to the actin filaments, shortening the muscle. Mutations in the genes of structural and regulatory muscle proteins can lead to the development of pathological conditions such as congenital myopathies and distal arthrogryposis in skeletal muscle, cardiomyopathies, and certain types of cancer. This Special Issue provides information about mutations in the genes of actin, tropomyosin, and Z-line proteins, all of which can lead to pathological changes in the muscles. Dysregulation of myoblast differentiation, impaired post-translational modification of proteins, and cell aging can also cause a contractility malfunction. The main aims of biology are to identify primary reasons for pathological conditions, to describe the cascade of molecular and cellular events and functional and structural variations in the different interactions inside the sarcomere, and to propose new effective approaches to the rehabilitation of sarcomere function on a molecular level.

This Special Issue provides a clear and concise review of the current knowledge on pathogenic variants in the key Z-disc proteins: α-actinin, filamin C, myopalladin, myotilin, telethonin, and the Z-disc alternatively spliced PDZ motif. K. Wadmore, A.J. Azad, and K. Gehmlich [1] summarize approximately 200 different pathogenic variants in six genes, explore their involvement in myopathy and cardiomyopathy, and provide informative tables with minor allele frequency on GnomAD to critically re-evaluate pathogenicity. The mechanisms of muscle dysfunction in Z-disc proteins remain unknown, and many interesting discoveries can be expected in the coming years.

An unexpected and interesting paper by C. Richter, D. Szczesna-Cordary et al. investigates the role of dysregulation of nine sarcomeric and cytoskeletal genes across 20 cancer lineages [2]. The investigation focused on the major myosin and actin genes and found that uterine cancers have the highest frequencies of amplification and overexpression of the gamma actin gene, ACTG1. The authors demonstrate that each of the four uterine cancer subtypes, mixed endometrial carcinomas, serous carcinomas, endometroid carcinomas, and carcinosarcomas, involves approximately ~20 % ACTG1 gene amplification or overexpression. The CRISPR-CAS9 gene deletion of ACTG1 has the most robust and consistent effects on uterine cancer cells relative to the 20 other lineages. It is proposed that ACTG1 regulates the fitness of uterine cancer cells by modulating cell-intrinsic properties and the tumor microenvironment. ACTG1 can be seen as a potential biomarker and a target gene in uterine cancer precision therapies. The authors’ findings indicate precision strategies against ACTG1 could yield benefits for a subset of uterine cancer patients with high-grade tumors.

Actin is highly conserved and ubiquitously expressed in all eukaryotic cells, and involved in more protein–protein interactions than any other known protein. It is not surprising, then, that changes in the structure and stability of actin relate to various dysfunctions. In muscle contraction, actin is often unjustly regarded as a relatively passive basis for sliding. However, actin filament is very dynamic, and its conformational state is critical for effective contraction. There are multiple mutations in actin responsible for nemaline myopathies; this forms the basis of the study by J. Gruszczynska-Biegala, S. Khaitlina, H. Strzelecka-Gołaszewska et al. [3]. The authors provide experimental evidence for the involvement of Segment 227–235 of actin in salt-induced stabilization of contacts within the actin filament and suggest that these contacts can be weakened by mutations characteristic of actin-related myopathies. Actin stabilization is observed at ionic, nearly physiological conditions and is weakened by the modification of the C-terminus as well as by myopathy-associated mutations.

Actin-related regulation is central to skeletal and cardiac muscles. The disturbance in the mechanisms of this type of regulation can be observed at the site of the mutations in a number of actin-binding proteins. The key regulation proteins are tropomyosin (Tpm) and troponin. The amino acid substitution Arg90Pro in Tpm3.12 due to the TPM3 gene mutation was found to be cause of congenital fiber-type disproportion and muscle weakness. In the work of Y. Borovikov, D. Andreeva, O. Karpicheva et al. [4], it was shown that such a defect affects actin–myosin interaction not only locally, but globally. The molecular mechanisms underlying muscle dysfunction in this disease include the abnormally high Ca^2+^ sensitivity of myofilaments, and depression of troponin’s ability to switch actin monomers off and reduce the number of myosin heads weakly bound to F-actin at low Ca^2+^. The effects of two chemical compounds in a single muscle fiber containing the Arg90Pro-mutant tropomyosin were evaluated. The data seem to indicate that BDM, an inhibitor of myosin ATPase activity, and W7, a troponin C antagonist, are able to restore tropomyosin’s capacity to engage in Ca^2+^-dependent movement and the ability of the troponin–tropomyosin complex to switch actin monomers off, demonstrating a weakening of the damaging effect of the Arg90Pro substitution on muscle contractility.

Congenital myopathies are linked to the mutations in genes of tropomyosin TPM2 и TPM3. In most cases, these mutations cause a hypocontractile muscular phenotype. In contrast, distal arthrogryposis is associated with mutations in TPM2 but not in TPM3 and demonstrates a hypercontractile phenotype. The effects of tropomyosin mutations with opposite phenotypes are compared in the research of J. Moraczewska [5]. It was shown that the presence of Gln93His (congenital myopathy) in homo- and heterodimers of tropomyosin strongly decreases activation of the actomyosin ATPase and reduces sensitivity of the thin filament to Ca^2+^. In contrast, the presence of Glu97Lys (distal arthrogryposis) causes hyperactivation of the ATPase and increases sensitivity to Ca^2+^. The data support the existence of several different mechanisms of muscle weakness which have yet to be described in detail.

The pathogenic effects of mutations in TPM can be caused not only by the local changes in structure but also those in the properties of sites situated at a significant distance from the point mutation. Unstable regions of tropomyosin are believed to play a significant part in protein–protein interactions. Experiments performed at the laboratory of D.I. Levitsky [6], in which a tropomyosin molecule was stabilized by substitutions Ala134Leu and Glu218Leu, show that these two regions—the central part and C-terminal region near the Glu218 residue—are necessary for proper tropomyosin binding to F-actin. An in vitro motility assay demonstrated that regions of instability are also essential for correct regulation of Ca^2+^-dependent interaction between myosin heads and F-actin.

It is not only genetic mutations in proteins that cause pathological conditions of the sarcomere, but also changes in the post-translational modification of proteins. Protein neddylation is a post-translational modification in which the ubiquitin-like molecule NEDD8 is covalently linked to specific lysine residues of target proteins. Neddylation regulates a broad range of substrates involved in diverse cellular processes. Neddylation has recently been implicated in many pathophysiological statuses. It was shown that inhibition of neddylation through a pharmacological blockade prevented terminal myoblast differentiation partially through repressing MYOG (myogenin) expression [7]. Mechanistically, neddylation deficiency enhances the mRNA and protein expressions of class IIa histone deacetylases, and prevents the downregulation and nuclear export of class III HDAC, all of which have been shown to repress MYOD1-mediated MYOG transcriptional activation. Therefore, neddylation plays an essential role in mediating myoblast differentiation and thus muscle cell mass and regeneration. Against the background of recent evidence supporting neddylation inhibitors as potential cancer therapeutics, this study provides insight into off-target effects.

Proper muscle function depends on the neuromuscular junction (NMJ), which generally transforms from simple oval plaques into “pretzel-like” structures. Structural transformation of the NMJ into an elaborate, perforated acetylcholine receptor array is pivotal for efficient synaptic transmission. Disrupted NMJ morphology has been reported in various myopathies, such as Duchenne muscular dystrophy, myasthenia gravis, congenital myasthenic syndromes, and spinal muscular atrophy. P. Alvarez-Suarez, M. Gawor et al. explored the role of drebrin—an actin and microtubule cross-linker—in postsynaptic machinery [8]. Drebrin is identified as a postsynaptic protein colocalizing with the postsynaptic acetylcholine receptors and is enriched at synaptic podosomes. The results reveal an interplay between drebrin and cluster-stabilizing machinery involving rapsyn, actin cytoskeleton, and microtubules. Downregulation of drebrin or the blocking of its interaction with actin in cultured myotubes impairs the organization of acetylcholine receptor clusters and the cluster-associated microtubule network.

Aging muscle cells undergo a series of changes that can also lead to cellular muscle dysfunctions. A. Moustogiannis, M. Koutsilieris et al. examined the effects of myoblast aging on skeletal myogenesis in vitro, using a multiple cell division model to replicate the cell senescence of C2C12 myoblasts [9]. The authors evaluated the alterations in the mRNA and/or protein expression of myogenic regulatory factors (MRFs), IGF-1 isoforms, apoptotic, atrophy, inflammatory, metabolic, and aging-related factors. Aged myoblasts exhibited G0/G1 cell cycle arrest, DNA damage, increased SA-β-gal activity and expression of aging-related factors p16 and p21 during differentiation, a reduction in the expression of MRFs and metabolic/anabolic factors, and an increased expression of apoptotic, atrophy, and inflammatory factors. A diminished differentiation capacity characterized the aged myoblasts, which, in combination with the induction of apoptotic and atrophy factors, indicates a disrupted myogenic lineage in the senescent muscle cells.

Since the inception of the theory of sliding, our knowledge has undoubtedly expanded with each such issue. Understanding the primary causes of muscle weakness and hypotension in cardiac and skeletal muscles in hereditary myopathies, leading to impaired muscle differentiation, aging, and associated functional consequences, is necessary for early diagnosis and prognosis of the dysfunction. However, despite the obvious progress in this field, many molecular mechanisms remain unresolved, and most importantly, we are on the verge of an era of abundant knowledge that can be used to restore the function of the sarcomere for the application of basic research in clinics.
